# Supplementary material for: Global DNA Methylation and mRNA-miRNA Variations Activated by Heat Shock Boost Early Microspore Embryogenesis in Cabbage (Brassica oleracea)
Source: Int J Mol Sci. 2022 May 5;23(9):5147. doi: 10.3390/ijms23095147 (PMC9103256; doi:10.3390/ijms23095147)
Supplement: Supplementary file 1 [file ijms-23-05147-s001.zip › ijms-1634790-supplementary/Supplementary Figures.pdf]

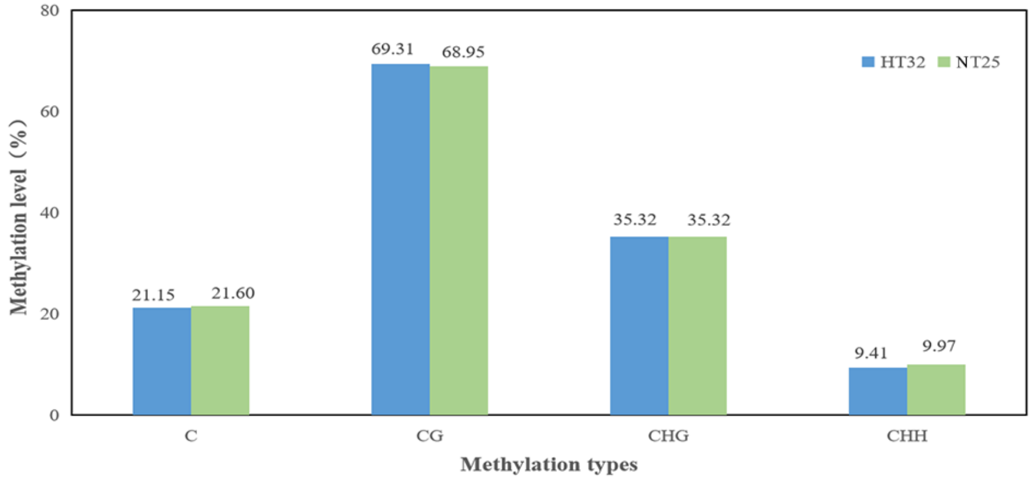

Figure S1. Levels of CG, CHG and CHH methylation in the whole cabbage genome.

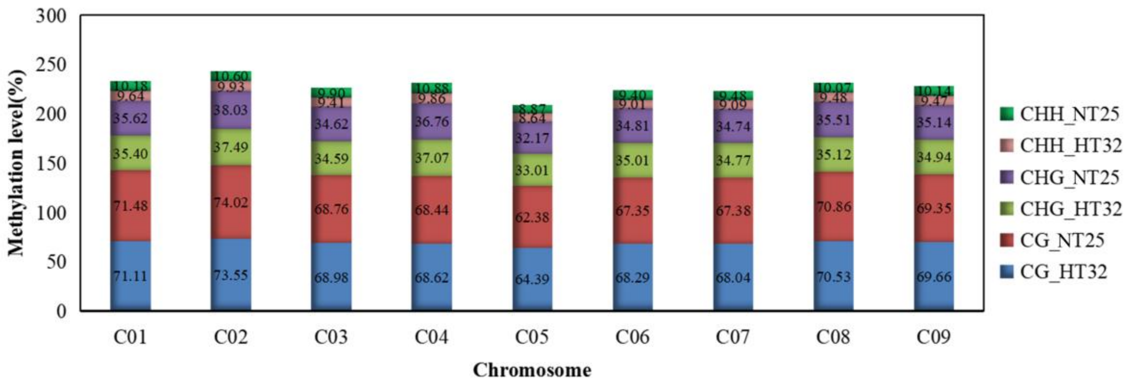

Figure S2. DNA methylation levels in nine chromosomes.

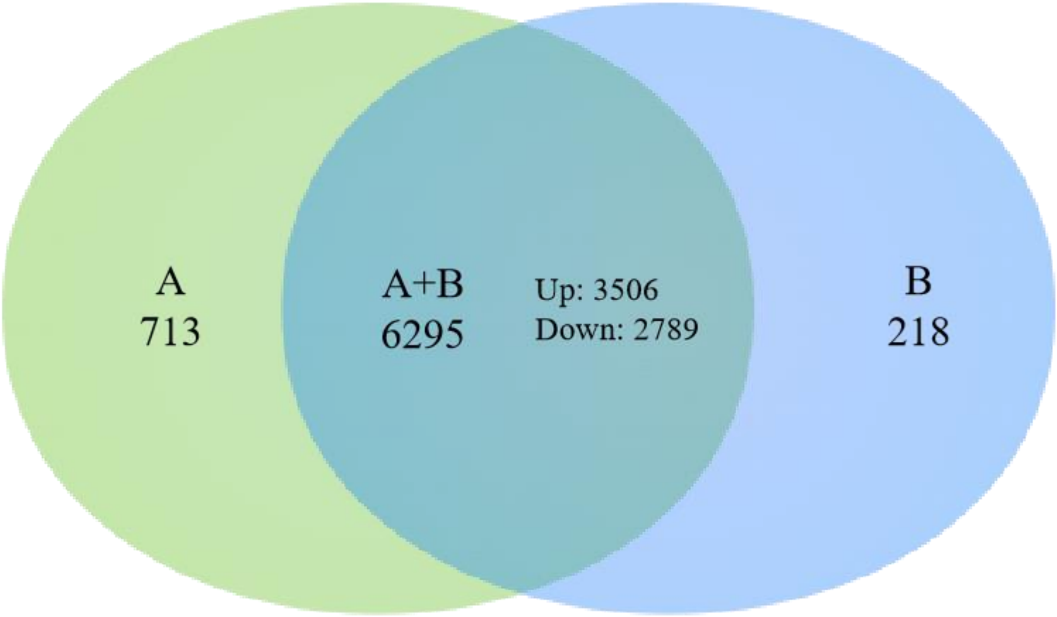

Figure S3. Ven diagram of DEGs in HT32 and NT25. A indicates the unique DEGs in HT32. B indicates the unique DEGs in NT25. A+B indicates the common DEGs in both samples.

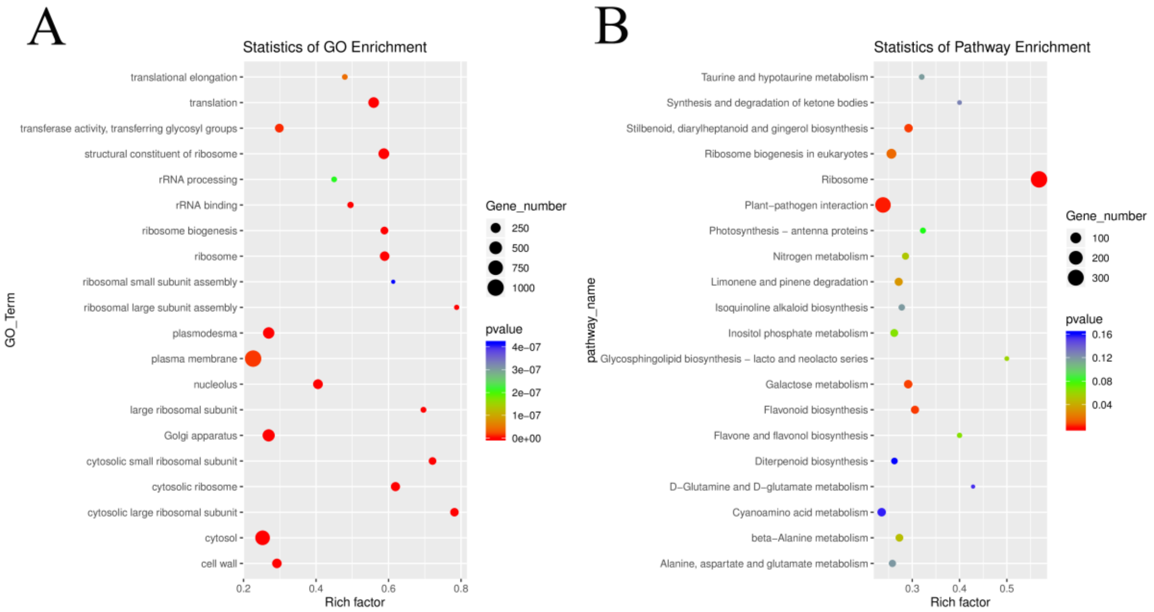

Figure S4. GO and KEGG enrichment of DEGs in microspores after HS. (A) GO enrichment of DEGs in microspores after HS. (B) KEGG enrichment of DEGs in microspores after HS.

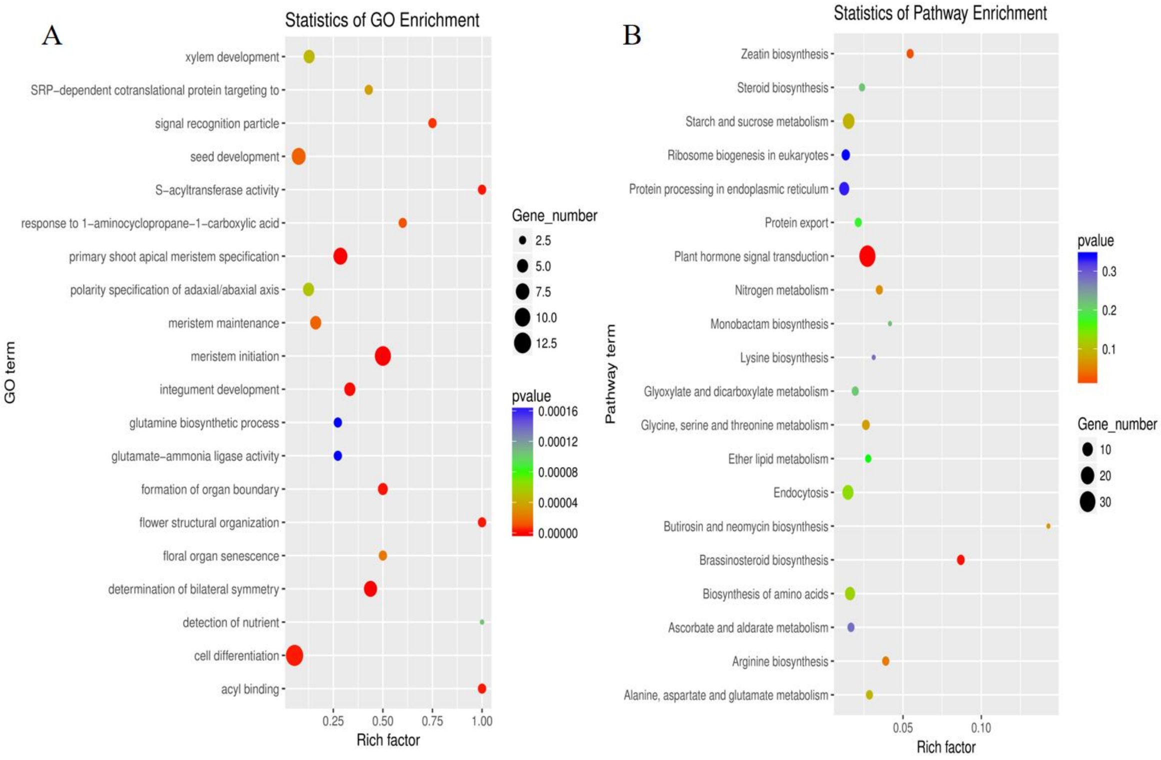

Figure S5. GO and KEGG enrichment of genes targeted by DERs. (A) GO enrichment of genes targeted by DERs. (B) KEGG enrichment of genes targeted by DERs.
